# Investigation on Dynamic Characteristics of AlGaN/GaN Lateral Schottky Barrier Diode

**DOI:** 10.3390/mi12111296

**Published:** 2021-10-22

**Authors:** Haitao Zhang, Xuanwu Kang, Yingkui Zheng, Hao Wu, Ke Wei, Xinyu Liu, Tianchun Ye, Zhi Jin

**Affiliations:** 1 High-Frequency High-Voltage Device and Integrated Circuits Center, Institute of Microelectronics of Chinese Academy of Sciences, Beijing 100029, China; zhanghaitao19@mails.ucas.edu.cn (H.Z.); zhengyingkui@ime.ac.cn (Y.Z.); wuhao@ime.ac.cn (H.W.); weike@ime.ac.cn (K.W.); xyliu@ime.ac.cn (X.L.); tcye@ime.ac.cn (T.Y.); 2 University of Chinese Academy of Sciences, Beijing 100029, China; 3 The Institute of Future Lighting, Academy for Engineering and Technology, Fudan University (FAET), Shanghai 200433, China

**Keywords:** GaN, SBD, Schottky barrier diode, simulation, current collapse, electric field, acceptor trap, conduction band energy

## Abstract

This work investigates the transient characteristics of an AlGaN/GaN lateral Schottky barrier diode (SBD) and its recovery process with a dedicated dynamic measurement system. Both static and dynamic characteristics were measured, analyzed with the consideration of acceptor/donor traps in the C-doped buffer and GaN channel, and verified by Silvaco TCAD (technology computer aided design) simulations. The energy band, electric field, and electron concentration were monitored in the transient simulation to study the origin of the current collapse in the SBD. Using the verified model, the impact of carbon doping concentration in the buffer and the thickness of the unintentionally doped (UID) GaN channel in the transient behavior was estimated. Several observations were revealed. Firstly, the traps in the GaN channel and buffer layer have a significant impact on the current collapse of the device. A severe deterioration of current collapse can be observed in the SBDs with increasing density of acceptor-like traps. Secondly, the current collapse increases with the thinner UID GaN channel layer. This well-performed simulation model shows promise to be utilized for the dynamic performance optimization of GaN lateral devices.

## 1. Introduction

A GaN-based Schottky barrier diode (SBD) is a promising device for next-generation electrical power systems, attributed to its superior material properties, such as high mobility, high electric breakdown strength, and high electron saturation velocity. Moreover, the process flow of the GaN SBD is compatible with GaN high electron mobility transistor (HEMT) and metal-insulator-semiconductor HEMT (MIS-HEMT), promising for its integration in the smart GaN platform. For a high-efficiency power system, a low turn on voltage and a high breakdown voltage are preferred. Various SBD designs and process techniques have been discussed to optimize Ni/Au based SBD, including conventional (non-recessed) SBD [1], partially recessed SBD [2,3], over-etched with sidewall contacted SBD [4], hybrid [5,6,7], and three-dimensional (3D) anode SBD [8,9]. However, most of the solutions proposed need an additional Schottky barrier “treatment”, e.g., recess or implantation, which might result in lattice damage in the Schottky region, leading to potential reliability problems [10,11].

In our previous work, a recess-free AlGaN/GaN heterojunction Schottky diode structure with a thin barrier was proposed, with a two dimensional electron gas (2DEG) being effectively preserved by silicon nitride (SiN_x_) passivation grown by low pressure chemical vapor deposition (LPCVD) [12,13]. We demonstrated the static characteristics of the SBD in our previous work, but dynamic characterization and quantitative modeling are still missing.

Most simulations of the dynamic characteristics of GaN HEMTs are on high conductive substrates (Si) [14,15,16], and the substrates of the devices are generally grounded or placed under a fixed negative bias voltage. When the electrical stress is applied, the substrate is at a fixed potential, thus forming a potential difference with the applied voltage on the surface of the devices. In this work, we mainly focus on physics-based simulation of the thin-barrier AlGaN/GaN SBD. Especially, the GaN has been epitaxially grown on a sapphire substrate in this work, which is highly resistive to be considered as a floating terminal. The substrate is also set as a float in the simulation process so as to study the influence of the electrical stress caused by the horizontal potential difference on the device.

In addition, there is a significant challenge to characterize the dynamic behaviors of GaN SBDs. The commonly used system to test the current collapse of GaN HEMT can only maintain the voltage and current in the same direction. Therefore, the current collapse test on the GaN SBD cannot be implemented with the conventional test system. An AccoTEST STS8200 tester has been modified to work bidirectionally, including a conventional static test on GaN SBD and current collapse, as well as achieving a voltage switch in a very short period of time.

SBDs with different anode and cathode spacing were fabricated for static and dynamic electrical measurement and simulation. The energy band, electric field, and electron concentration were monitored in the transient simulation to study the origin of the current collapse in the SBD. Using the verified model, the impact of carbon doping concentration in the buffer and the thickness of the UID GaN channel on the dynamic behavior was studied. Several observations were revealed. Firstly, the current collapse is deeply dependent on the traps in the GaN channel and the buffer layer. A severe deterioration of the current collapse and a higher recovery rate can be observed in the SBDs with increasing density of acceptor-like traps. Secondly, a more severe current collapse is observed with a decrease in the thickness of the UID GaN channel layer.

## 2. Device Characterization and Simulation

**Figure 1:**
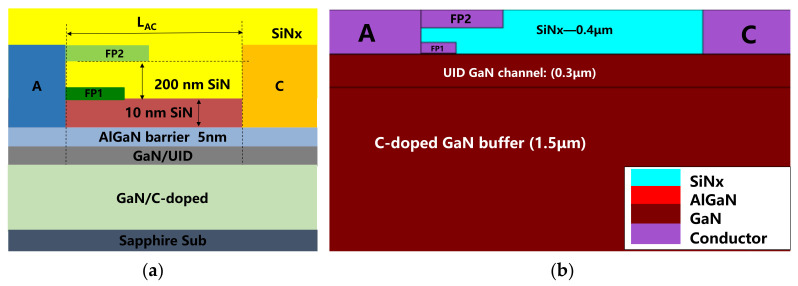
(**a**) Schematic of the Schottky barrier diode (SBD) for its measurement. (**b**) Schematic of the SBD for its simulation.

The schematic of the SBD is shown in Figure 1. The AlGaN/GaN heterostructure epitaxial wafer starts in a 4 inch sapphire substrate by metal organic chemical vapor deposition (MOCVD), consisting of a ~1.5 μm C-doped GaN buffer stack, a 300 nm GaN channel, a 1 nm AlN interface enhancement layer, and a 5 nm Al_0.25_Ga_0.75_N barrier. On top of the AlGaN barrier is a 10 nm LPCVD SiNx, which is under the first layer field plate (FP1), and another 200 nm SiNx is in between the FP1 and FP2.

**Figure 2:**
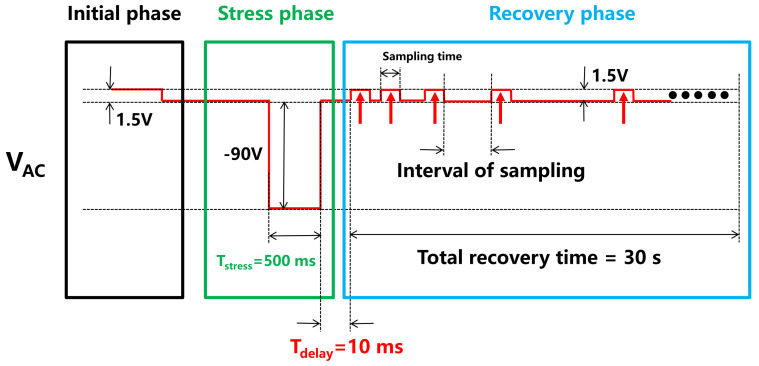
Dynamic measurement test sequence with an STS8200.

The dynamic measurement sequence is shown in Figure 2. In the first phase, the initial forward current (I_AC_) was measured with an STS8200 at V_AC_ = 1.5 V before the stress. In the second phase, the total stress time and the stress voltage were 500 ms (T_stress_) and 90 V (V_AC_ = −90 V), respectively. In the third phase, the recover current I_AC_ (@V_AC_ = 1.5 V) was measured immediately after the stress with a delay time (T_delay_) of 10 ms. During the recovery phase, the current was measured in a pulsed mode with a short sampling time of 20 μs to avoid self-heating. In addition, the interval between each sampling was gradually increased with a total recovery time of 30 s. In the simulation for dynamic characteristics, the test sequence and settings were identical to the electrical measurement, except with a constant of 1.5 V in the recovery phase.

The work function of TiN is reported mostly as 4.6–5.0 eV [17,18,19]. Therefore, the work function of the Schottky metal was set at 4.65 eV to obtain a good fitting on the electrical measurement and the Silvaco TCAD (technology computer aided design) simulation. In addition, the value of polarization charge density in the access region, where the AlGaN barrier is passivated by SiNx, was set at $1.35\times 10^{13}$ cm^−2^ by default. The 2DEG sheet charge density of the thin AlGaN/GaN barrier was very low in the area without SiNx passivation, thereby the value of polarization charge density in the Silvaco simulation under the Schottky metal was set at $2.5\times 10^{12}\;$cm^–2^ [20,21]. The ohmic contact-related parameter in the simulation was set at 0.5 Ω mm, which is consistent with our test results.

Regarding TCAD simulation, it is widely acknowledged that the UID GaN exhibits an n-type conductivity with incomplete ionization of donors and a modest leakage [22], whereas the carbon compensation doping in the buffer layer can induce deep-level acceptor traps [23]. It has been mentioned in the literature [24] that the energy level of E_C_ −1.10 eV is measured in GaN, and the energy level of E_V_ +0.9 eV caused by carbon doping has been confirmed in many instances in the literature [14,15,16,25,26]. Therefore, a donor trap of Ec −1.02 eV in the UID GaN layer and an acceptor trap of Ev +0.81 eV [14,15] in the C-doped buffer layer were set in the simulation. Both the electron and hole capture cross sections for the deep donor and acceptor traps were set as $1\times 10^{-13}$ cm^2^. It is necessary to set the energy levels carefully, because the capture and emission of the traps are expressed by exponential functions of the energy levels, so any slight change in the energy levels will greatly affect the results. Finally, the electrical measurement and simulation are well fitted. Table 1 summarizes the key parameters of simulation.

**Figure 3:**
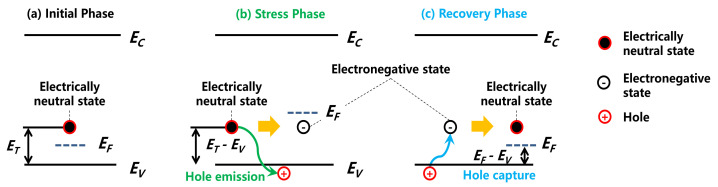
Schematic of acceptor-trap-induced capture and emission of free carriers.

The charge trapping effects can influence the response time of capture and emission of free carriers. Taking the deep-level acceptor traps in the GaN buffer layer as an example, in the stress phase (Figure 2), the acceptor traps release holes under a high electric field, yielding negative space charges in the buffer layer [25,26,27,28], as shown in Figure 3b. The hole emission rate can be expressed as shown in Equation (1):

(1) $$e_{p}\;=\sigma_{p}\nu_{p}N_{V}e^{-\frac{E_{T}-E_{V}}{kT}}$$

where $\sigma_{p}\;$is the capture cross section of the acceptor trap; $\upsilon_{p}$ is the thermal velocity of the hole; $N_{V}$ is the valence band state density; and $E_{T}$ is the acceptor trap level.

In the recovery phase (Figure 2), these negatively charged acceptor traps start to capture holes from the valence band, resulting in a GaN buffer layer that is electrically neutral, as shown in Figure 3c. The hole capture rate can be expressed as shown in Equation (2):

(2) $$c_{p}=\sigma_{p}\nu_{p}p=\sigma_{p}\nu_{p}N_{V}e^{-\frac{E_{F}-E_{V}}{kT}}$$

where p is the hole density and $E_{F}-E_{V}$ is the difference between the Fermi energy level and the valence band. In Equation (2), since $c_{p}$ increases with the increase in temperature, the recovery process can be accelerated at elevated temperatures. It can be seen from Equations (1) and (2) that the hole emission rate is determined by the trap energy level, and the hole capture rate is determined by the Fermi level (i.e., the hole concentration in the valence band), as shown in Figure 3.

## 3. Results and Discussion

**Figure 4:**
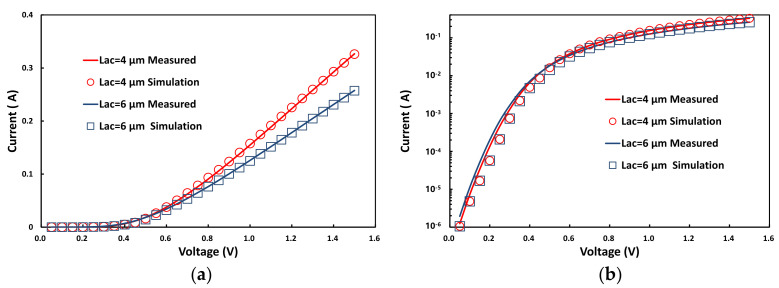
(**a**) Static current-voltage characteristics in linear coordinates. (**b**) Static current-voltage characteristics in logarithmic coordinates.

The static and dynamic characteristics were carried out for different L_AC_ (4 μm and 6 μm). As shown in Figure 4, the measured I-V curve is in concordance with the simulated I-V curve and slightly differs for Log(I)-V curves.

**Figure 5:**
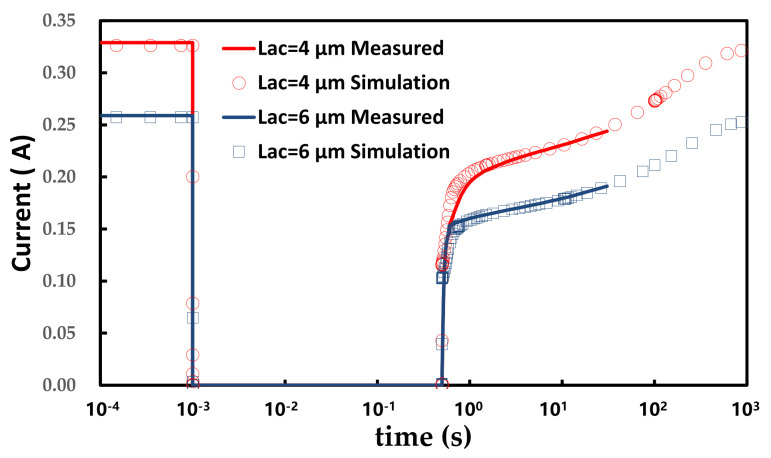
Dynamic measurement and simulations.

In addition to the static current-voltage measurement, the transient measurement was carried out on GaN SBDs with L_AC_ = 4 μm and L_AC_ = 6 μm. In the first phase (10^−4^ s to 10^−3^ s), the initial forward current (I_AC_) was measured at V_AC_ = 1.5 V. In the stress phase (10^−3^ s to 500 ms), the stress voltage was 90 V (V_AC_ = −90 V). In the recovery phase, the forward current I_AC_ (@V_AC_ = 1.5 V) was measured immediately after the stress with a delay time (T_delay_) of 10 ms. The measured result and simulation with the model mentioned in the Section 2 are shown in Figure 5 with solid lines and circles, respectively. The current gradually increased, attributed to the slow capture of the holes in the C-doped buffer layer, leading to an increase in the 2DEG sheet charge density. The measured result is in accordance with the simulation model.

**Figure 6:**
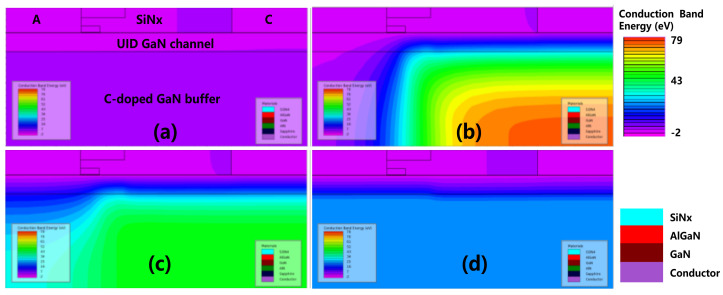
The conduction band energy distribution at different timings. (**a**) t_0_: the initial state; (**b**) t_1_; (**c**) t_2_; and (**d**) t_3_.

Figure 6 shows the conduction band energy distribution diagram when V_AC_ = 1.5 V at different timings; (a) t_0_ is the initial state without electrical stress; (b) t_1_ is 100 μs after the electrical stress; (c) t_2_ is 100 ms after the electrical stress; and (d) t_3_ is 100 s after the electrical stress. In Figure 6b, the conduction band energy is lifted up significantly after the reverse stress. In Figure 6c,d, the conduction band is gradually lowered and recovered after a sufficient time delay.

**Figure 7:**
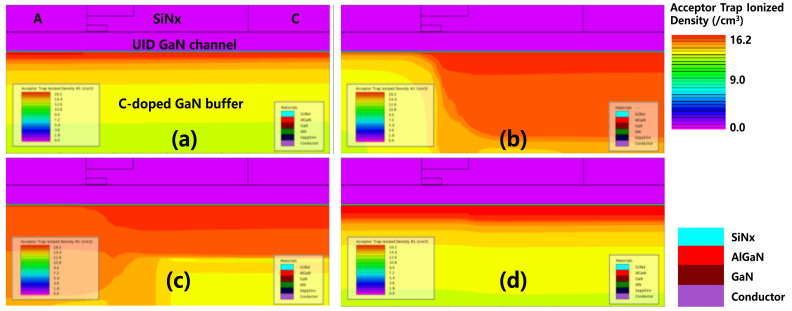
Ionization concentration of the acceptor traps distribution at different timings. (**a**) t_0_: the initial state; (**b**) t_1_; (**c**) t_2_; and (**d**) t_3_.

Figure 7 illustrates the ionized acceptor trap concentration when V_AC_ = 1.5 V at different timing; (a) t_0_ is the initial state without electrical stress; (b) t_1_ is 100 μs after the electrical stress; (c) t_2_ is 100 ms after the electrical stress; and (d) t_3_ is 100 s after the electrical stress. The concentration of the ionized acceptor traps in the C-doped buffer layer was high just after the reverse stress at t_1_. The concentration of the ionized acceptor traps gradually decreased and recovered towards the neutral state with the decrease in conduction band energy level.

**Figure 8:**
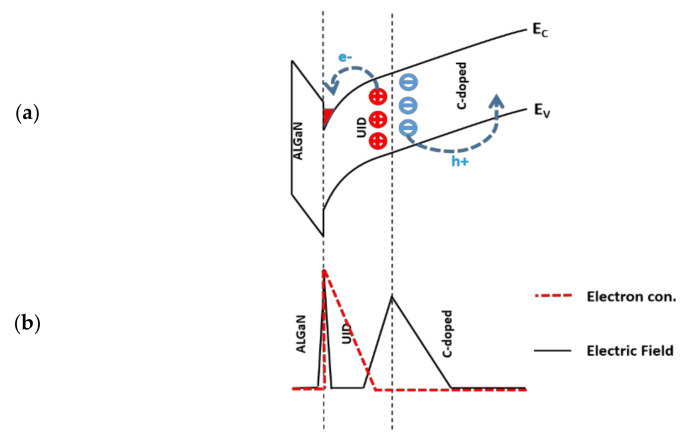
(**a**) Schematic band diagram under V_stress_, showing the ionization of donor and acceptor traps at the unintentionally doped (UID)/C-doped GaN junction. (**b**) Electric field and electron concentration under V_stress_ at the UID/C-doped GaN junction.

V_stress_ applied to the SBD yielded negative charges in the buffer and positive charges (or reduction of electrons) in the 2DEG. As shown in Figure 8, the opposite doping polarities in the UID and C-doped buffer layers could result in a *p*–*n* junction at the UID/buffer interface, which becomes reversely biased under V_stress_ with the depletion region extending into the two layers and excessive ionization of both donor and acceptor traps [13]. Moreover, the ionization of the acceptor traps in the buffer layer can result in negative space charges, leading to a significant reduction of the 2DEG sheet charge density and forward current. The higher V_stress_ or higher acceptor trap concentration in the buffer layer can yield a higher density of negative charges, causing a severe current collapse phenomenon. Likewise, the ionization of donor traps can yield positive charges, which can compensate the negative charges generated in the buffer layer, resulting in less depletion of the 2DEG under V_stress_.

**Figure 9:**
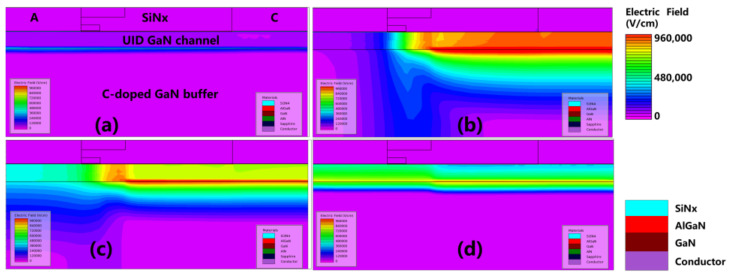
The electric field distribution at different timings. (**a**) t_0_: the initial state; (**b**) t_1_; (**c**) t_2_; and (**d**) t_3_.

In Figure 9a, the initial state of the SBD without reverse stress is shown. Figure 9b–d shows the electric field distribution at different timings after stress with t_1_, t_2,_ and t_3_, respectively. At t_1_, the electric field at the interface between UID and C-doped buffer layer increases, leading to a significant increase in the electric field in the GaN channel. At t_2_ and t_3_, the electric field gradually decreases with the decrease in the conduction band energy level.

**Figure 10:**
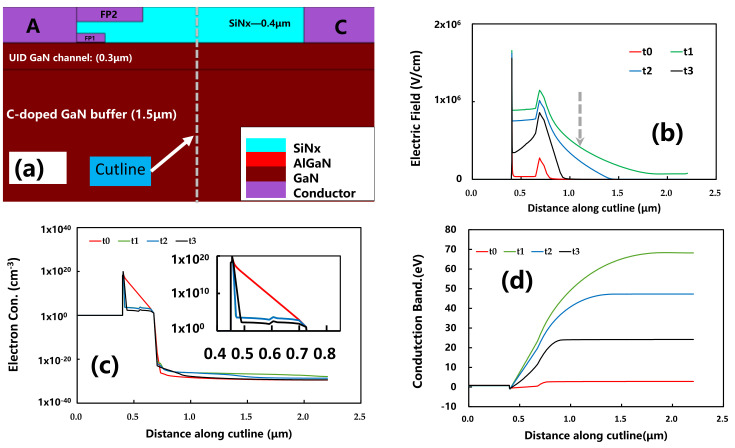
Schematic of (**a**) the cutline location; (**b**) the electric field at different timings; (**c**) the electron concentration in the channel region at different timings; and (**d**) the conduction band energy at different timings.

Figure 10 shows the conduction band energy, electric field, and electron concentration at t_0_~t_3_ from the top surface to the bottom layer in the vertical direction of the device access region (the cutline schematic is shown in Figure 10a). It can be clearly seen in the channel region that the electric field gradually decreases and recovers towards the initial state in t_0_, as shown in Figure 10b. The electron concentration in the 2DEG gradually increases and closes to the initial state in t_0_ in Figure 10c. The concentration of ionized acceptor traps increases after the electrical stress, resulting in an increase in conduction band energy, electric field in the UID GaN channel, and reduction of the electron concentration in the UID GaN channel, eventually causing the current collapse phenomenon.

**Figure 11:**
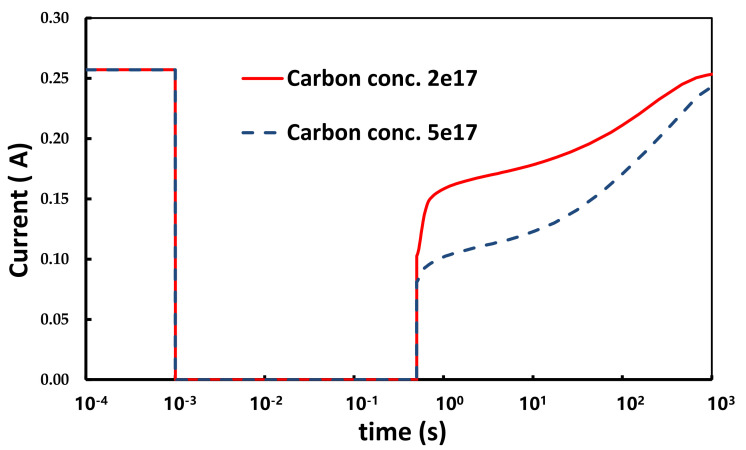
The transient current for the C-doping concentration at $2\times 10^{17}$ cm^−3^ and $5\times 10^{17}$ cm^−3^ in the buffer after stress.

**Figure 12:**
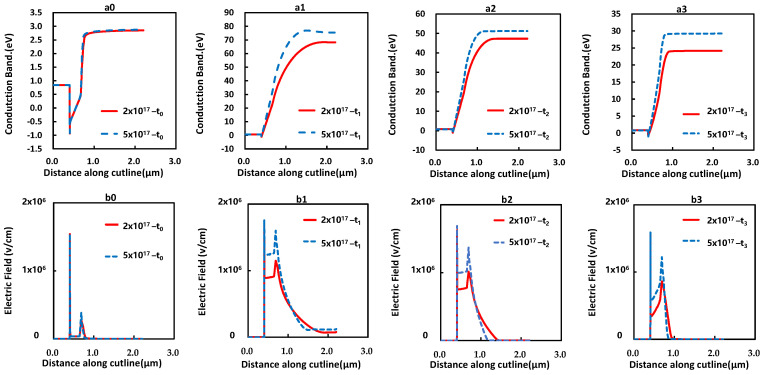
(**a_0_**–**a_3_**). The curve of the conduction band of the device with different carbon doping concentrations at different moment t**_0_**~t**_3_**; (**b_0_**–**b_3_**). The electric field of the device with different carbon doping concentrations at moments t**_0_**~t**_3_**.

Based on the model suggested above, the impact of the carbon doping concentration and the thickness of UID layer on the transient current was simulated and studied. The simulation result with increased carbon doping concentration in the C-doped buffer layer from $2\times 10^{17}$ cm^−3^ to $5\times 10^{17}$ cm^−3^ is shown in Figure 11. The current collapse at 100 μs was drastically increased and the recovery rate towards the initial current as a fresh device was evidently faster. For different carbon doping concentrations, the conduction band energy and electric field of devices at different timings was simulated and is shown in Figure 12a,b, which was along the cutline as shown in Figure 10a. For the higher carbon doping device ($5\times 10^{17}$ cm^−3^), the higher electric field, the conduction band energy in the UID GaN channel region, and the more severe current collapse were observed. Furthermore, the higher electric field intensity at the UID/buffer interface was observed.

According to Equation (1), the traps have the same capture rate of free carriers with the same trap levels, stress voltage, and stress time. Therefore, a higher trap concentration will allow more free carriers to be released, which will lead to a severe current collapse. From Equation (2), it can be concluded that the capture of free carriers by traps is mainly determined by the Fermi level, which depends on hole concentration. The higher acceptor trap concentration has the higher capture rate, leading to a faster recovery rate towards the initial current level.

**Figure 13:**
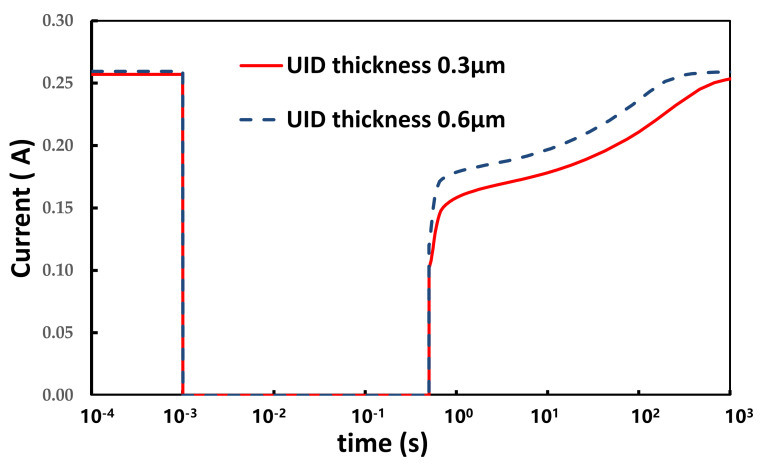
Current collapse for UID thicknesses of 0.3 μm and 0.6 μm.

**Figure 14:**
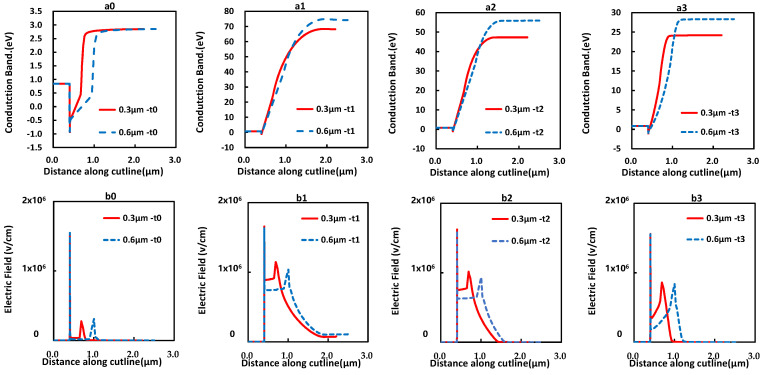
(**a_0_**–**a_3_**) The conduction band of the device with different UID GaN channels at moments t**_0_**~t**_3_**. (**b_0_**–**b_3_**) The electric field of the device with different UID GaN channels at moments **t_0_**~**t_3_**.

In order to reduce the problem of the current collapse of the device, we assumed that the average electric field needed to be reduced in the UID GaN layer. Based on this assumption, therefore, the impact of the UID GaN layer thickness was studied and verified by the simulation. As shown in Figure 13, the device’s initial forward current at V_AC_ = 1.5 V is slightly higher, meanwhile the current collapse is significantly reduced, with the UID GaN layer thickness increased from 0.3 μm to 0.6 μm. This result is well agreed with the assumption. To explain the mechanism for the reduction of the current collapse, the conduction band energy and electric field of devices at different timings were simulated for different UID GaN layer thicknesses, as shown in Figure 14a,b, which was along the cutline as shown in Figure 10a. Simultaneously, lower average electric field intensity in the channel region with thicker UID was observed. As a result, the thicker UID can help to reduce the current collapse problem.

Chevtchenko et al. [29,30] found a tradeoff between the breakdown voltage and dynamic R_ON_, both of which show a strong dependence on the carbon doping concentration and the thickness of the UID GaN channel on top of the C-doped GaN buffer. It has been observed that the buffer structure with a higher carbon doping concentration and a thinner UID GaN channel on top of the C-doped GaN buffer can yield a higher breakdown voltage, but a worse dynamic performance, which is consistent with the observations and models in our paper.

## 4. Conclusions

Dynamic characteristics on a lateral AlGaN/GaN SBD was successfully carried out with a modified AccoTEST STS8200 tester. Both static and dynamic characteristics are well fitted by Silvaco TCAD simulation. Based on the well-verified model, predictive simulations were carried out further. Using the verified model, the impact of carbon doping concentration in the GaN buffer and the thickness of the UID GaN channel on the dynamic behavior was studied. Several observations were revealed. Firstly, the traps in the GaN channel and the buffer layer have a significant impact on the current collapse of the device. A severe deterioration of the current collapse can be observed in the SBDs with an increasing density of acceptor-like traps. Secondly, current collapse increases with a thinner UID GaN channel layer. This well preformed simulation model shows promise to be utilized for the dynamic performance optimization of GaN lateral devices.

## Figures and Tables

**Table 1:** Parameters utilized in the simulation.

Parameters	Value	Unit
Schottky metal work function	4.65	eV
Polarization charge density in the access region	$1.35\times 10^{13}$	cm^−2^
Polarization charge density in the electrode region	$2.5\times 10^{12}$	cm^−2^
C-doping concentration in the buffer	$2\times 10^{17}$	cm^−3^
E_T_ of the acceptor trap in the C-doped buffer	Ev +0.81	eV
E_T_ of the donor trap in the UID GaN channel	Ec −1.02	eV
Electron capture cross sections	$1\times 10^{-13}$	cm^2^
Hole capture cross sections	$1\times 10^{-13}$	cm^2^
